# Potential disease biomarkers for diabetic retinopathy identified through Mendelian randomization analysis

**DOI:** 10.3389/fendo.2023.1339374

**Published:** 2024-01-11

**Authors:** Xuyan Zou, Suna Ye, Yao Tan

**Affiliations:** ^1^ Changsha Aier Eye Hospital, Aier Eye Hospital Group, Changsha, China; ^2^ Senzhen Aier Eye Hospital, Jinan University, Shenzhen, China; ^3^ Department of Ophthalmology, The Third Xiangya Hospital, Central South University, Changsha, China; ^4^ Postdoctoral Station of Clinical Medicine, The Third Xiangya Hospital, Central South University, Changsha, China

**Keywords:** diabetic retinopathy, Mendelian randomization, genomics, proteomics, disease biomarkers

## Abstract

**Background:**

Diabetic retinopathy (DR), a leading cause of vision loss, has limited options for effective prevention and treatment. This study aims to utilize genomics and proteomics data to identify potential drug targets for DR.

**Methods:**

We utilized plasma protein quantitative trait loci data from the Atherosclerosis Risk in Communities Study and the Icelandic Decoding Genetics Study for discovery and replication, respectively. Genetic associations with DR, including its subtypes, were derived from the FinnGen study. Mendelian Randomization (MR) analysis estimated associations between protein levels and DR risk, complemented by colocalization analysis to examine shared causal variants.

**Results:**

Our MR analysis identified significant associations of specific plasma proteins with DR and proliferative DR (PDR). Elevated genetically predicted levels of WARS (OR = 1.16; 95% CI = 0.095-0.208, FDR = 1.31×10^-4^) and SIRPG (OR = 1.15; 95% CI = 0.071-0.201, FDR = 1.46×10^-2^) were associated with higher DR risk, while increased levels of ALDOC (OR = 1.56; 95% CI = 0.246-0.637, FDR = 5.48×10^-3^) and SIRPG (OR = 1.15; 95% CI = 0.068-0.208, FDR = 4.73×10^-2^) were associated with higher PDR risk. These findings were corroborated by strong colocalization evidence.

**Conclusions:**

Our study highlights WARS, SIRPG, and ALDOC as significant proteins associated with DR and PDR, providing a basis for further exploration in drug development. Additional studies are needed to validate these proteins as disease biomarkers across diverse populations.

## 1 Introduction

Diabetic retinopathy (DR) is a leading cause of vision loss worldwide, significantly impacting both individual health and public healthcare systems (1). Approximately one-third of people with diabetes develop severe visual impairments due to DR, leading to a substantial proportion suffering from irreversible blindness (2). The progression of DR is primarily driven by prolonged hyperglycemia, oxidative stress, and inflammation, resulting in microvascular damage in the retina (3). Current treatments, particularly anti-vascular endothelial growth factor (anti-VEGF) therapies, have marked a significant advancement in managing DR, effectively slowing its progression in many patients. However, about 40% of patients either resist or respond inadequately to these treatments, underscoring the need for more diverse and effective therapeutic strategies (4). Alongside anti-VEGF, other treatments, including steroid therapies and combination protocols, have been explored (5, 6). While these have shown limited efficacy, they represent important attempts in the ongoing effort to combat DR.

Circulatory proteins have become key targets for therapeutic research in DR, playing critical roles in various molecular processes (7). Previous studies have highlighted several proteins associated with the development of DR, including C-C motif chemokine 5 (CCL5), α-2-antiplasmin (SERPINF2), various adhesion molecules, and C-reactive protein (CRP) (8–10). These findings are significant in understanding the pathogenesis of DR and offer potential avenues for treatment. Advancements in high-throughput proteomics have further enriched our understanding of DR at the molecular level. For example, research by Lu et al. compared plasma proteomes of DR patients and identified key biomarkers like afamin and protein arginine N-methyltransferase 5, which are linked to the progression and development of diabetes (11). Similarly, Gopalakrishnan et al. discovered distinct protein expression profiles between DR and proliferative DR (PDR), with neuroglobin (NGB) standing out as a notable marker for DR development (12). However, these associations, primarily derived from observational studies, necessitate rigorous validation. This is crucial to ensure that the identified protein associations with DR are not confounded by external variables or biased by reverse causality. The pursuit of this validation represents a critical step in translating these proteomic discoveries into practical therapeutic interventions for DR.

Mendelian randomization (MR) utilizes single nucleotide polymorphisms (SNPs) from genome-wide association studies (GWAS) to uncover causal links between genetic factors and health outcomes (13). This method capitalizes on the random distribution of genes at birth, which helps overcome biases and confounding factors often encountered in observational studies (13). MR’s integration of advanced genomic and proteomic data has been instrumental in identifying potential disease biomarkers for various diseases (14, 15). Despite its proven utility, the application of MR in DR research remains limited. There is a significant opportunity to expand this approach in DR, particularly by combining insights from GWAS and protein quantitative trait loci (pQTL) datasets. This integration could offer new perspectives and solutions in understanding and treating DR, an area where there is still much to explore and discover.

Our study embarks on a comprehensive proteome-wide MR analysis, augmented by colocalization analysis, to explore potential disease biomarkers for DR and PDR. By integrating genomic and proteomic data, we aim to uncover new pathways and targets, potentially paving the way for innovative treatments for these visually debilitating conditions.

## 2 Materials and methods

### 2.1 Study design and ethics

**Figure 1:**
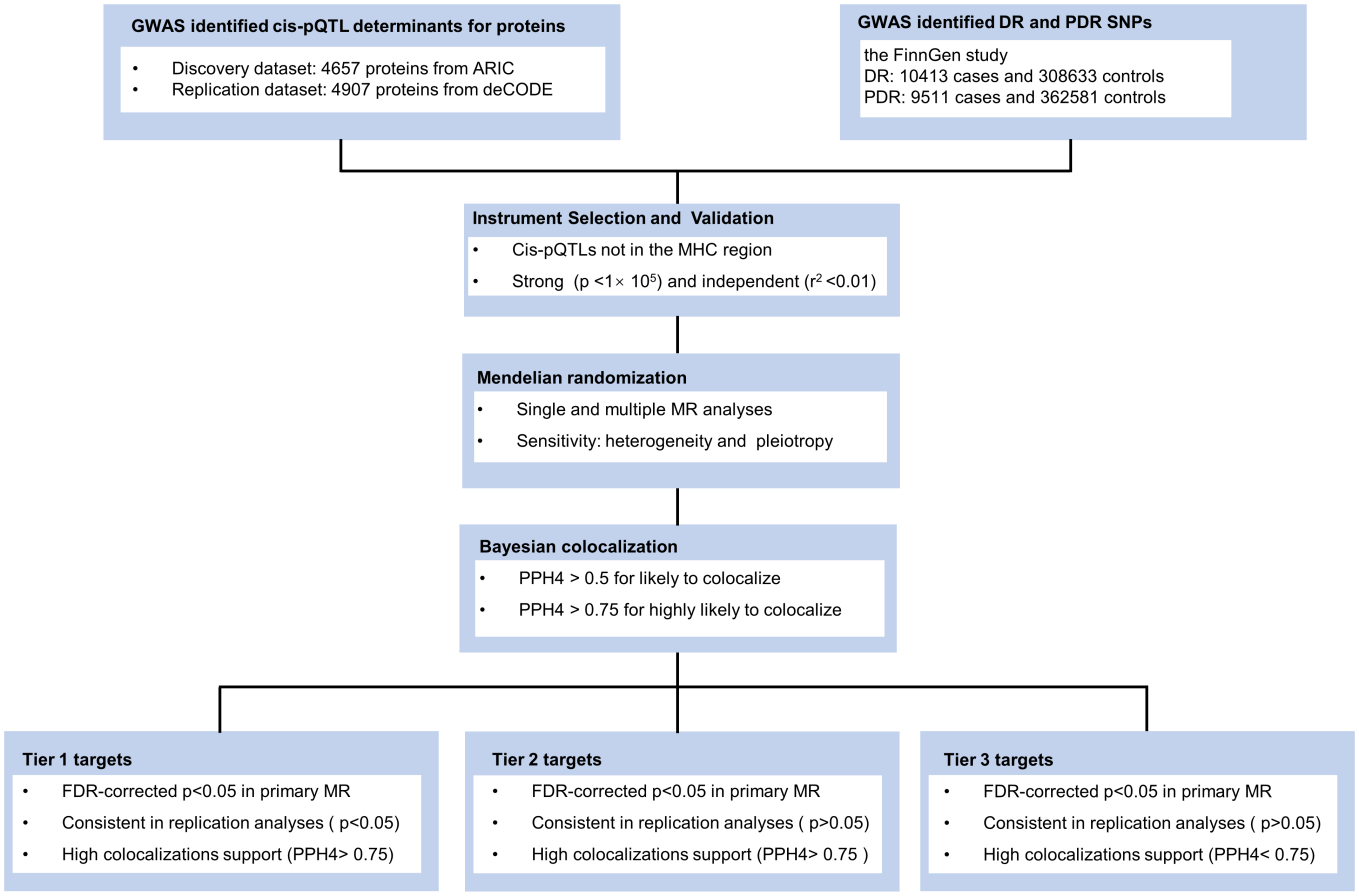
Study design. ARIC, the Atherosclerosis Risk in Communities study; deCODE, the Icelandic Decoding Genetics Study; DR, diabetic retinopathy; PDR, proliferative diabetic retinopathy; FDR, false discovery rate; MR, Mendelian Randomization.

Our research methodology is outlined in **Figure 1**. We utilized data from three key sources: the large-scale genome-wide blood proteome study (available at https://www.decode.com/summarydata/) (16), the Atherosclerosis Risk in Communities (ARIC) study (http://nilanjanchatterjeelab.org/pwas/) (17), and the FinnGen study (https://www.finngen.fi/en) (18). All the data were sourced from established studies that had already obtained ethical clearance from their respective institutions, eliminating the need for a separate ethical review for our research.

### 2.2 Data sources

In the discovery stage, plasma protein pQTL data were obtained from the ARIC study, which included a total of 4657 plasma proteins collected from 7213 European Americans (EA) (17). For the replication stage, plasma protein pQTL data were obtained from the Icelandic deCODE genetics study by Ferkingstad et al, which analyzed 4907 plasma proteins from 35,559 Icelanders and reported more than 272 million genetic variants (16). The use of both datasets allowed us to balance the discovery and validation phases of our study effectively. The ARIC dataset provided a detailed platform for initial protein association findings, while the Icelandic dataset enabled us to replicate and validate these findings across a different population, thereby enhancing the generalizability of our study. Proteomics analyses for both studies were performed using the advanced SomaScan technology on the v.4.1 platform, ensuring consistent and high-quality data for our analyses.

We obtained DR and PDR data from the FinnGen study (18). This included 10,413 DR cases and 308,633 controls, and 9,511 PDR cases and 362,581 controls. The participants were all of European descent. We adjusted genetic associations for factors like age, sex, and genetic correlation, along with genotyping batch and the top 10 principal components. We identified cases of DR and PDR using International Classification of Diseases codes, specifically ICD-9 (3620) and ICD-10 (H360) for DR, and ICD-10 (H3603) for PDR (18).

### 2.3 Instrument selection and validation

In conducting our MR analysis, we rigorously derived genetic instrumental variables (IVs) from plasma protein pQTL data, as referenced in our prior research (16, 17). To ensure a substantial association between these IVs and the exposure variable, which in our study is protein abundance, we meticulously selected cis-SNPs positioned within 1 megabase (Mb) of the gene encoding the relevant protein. This selection was based on a stringent p-value threshold of less than 1 × 10^-5^, a criterion chosen to balance statistical significance with the likelihood of a genuine biological impact on protein levels. To secure the independence of these SNPs and preclude confounding due to linkage disequilibrium, we employed linkage disequilibrium (LD) clumping using PLINK software. This process involved evaluating SNPs within a 10 Mb window, considering them as independent if their LD values (r^2^) was less than 0.01. Our choice of reference panel for this analysis was the genotype data of Europeans from the 1000 Genomes Project, which aligns with the ancestry of the study’s participant population. This methodological approach was adopted to ensure the reliability and relevance of the instrumental variables used in our MR analysis.

### 2.4 Mendelian randomization

All MR analyses were undertaken with the TwoSampleMR package in R. The primary MR analysis was conducted using the inverse-variance weighted (IVW) method to determine the causal effects of plasma proteins on DR. FDR correction was performed using the BH method, and FDR < 0.05 was considered for statistical significance. The MR results were presented as odds ratio (OR) and 95% confidence interval (95% CI) for risk of DR per genetically predicted 1-standard deviation (SD) increase in plasma protein level.

To further identify the associations identified by the primary analyses, we performed multiple MR analyses of the preliminarily identified proteins as replications using an independent blood pQTL database (The Icelandic deCODE genetics study). Multiple MR analytical approaches, including IVW, Egger, weighted median, and weighted mode, were applied for validation, of which IVW was chosen as the primary approach according to the recommendation (19).

To further assess the robustness of the causal relationships identified by the multiple MR analyses, we also conducted sensitivity analyses, including heterogeneity and horizontal pleiotropy tests. Heterogeneity was assessed using Cochran’s Q statistic (20). The Cochran’s Q test followed a chi-square distribution with IV number minus one degree of freedom. MR-Egger regression intercept was employed as a measure of directional pleiotropy (20). Proteins with only one IV are not suitable for the above sensitivity analyses. Heterogeneity tests require at least two IVs to be analyzed and MR-Egger regression requires at least three IVs to be analyzed. For the multiple MR and sensitivity analyses, p-value < 0.05 was considered significant.

### 2.5 Bayesian colocalization

Colocalization analysis serves as an indispensable complement to cis-MR, crucial for assessing the validity of IV assumptions (21). This analysis is pivotal in differentiating whether the same genetic variants are influencing both the exposure (plasma protein levels) and the outcome (DR risk). By conducting colocalization analysis using the coloc.abf function in the R package coloc, we aimed to determine if the identified proteins and DR share causal genetic variants within the same genomic regions. This step is crucial for eliminating potential interference due to LD. We tested the posterior probability of hypothesis 4 (PPH4), which examines the likelihood of both the protein and DR sharing variants in the same region. Interpretation of PPH4 values is critical; a PPH4 greater than 0.5 suggests a likely colocalization, while a value exceeding 0.75 indicates a high probability of sharing causal variants.

The results of the colocalization analysis were instrumental in categorizing the identified proteins into tiers based on the strength of their causal evidence with DR. Proteins that showed consistent results in replication analyses and had strong supporting evidence of colocalization (PPH4 greater than 0.75) were classified as Tier 1 targets. This classification underscores a robust association with DR, suggesting a higher likelihood of being genuine disease biomarkers. Proteins with only high support evidence of colocalization (PPH4 greater than 0.75) were categorized as Tier 2 targets. These proteins, while showing potential association with DR, may require further validation. The remaining proteins, which did not meet these stringent criteria, were classified as Tier 3 targets. This tier-based system allows for a nuanced interpretation of the data, guiding future research and development efforts towards the most promising disease biomarkers for DR.

## 3 Results

### 3.1 MR analysis

**Figure 2:**
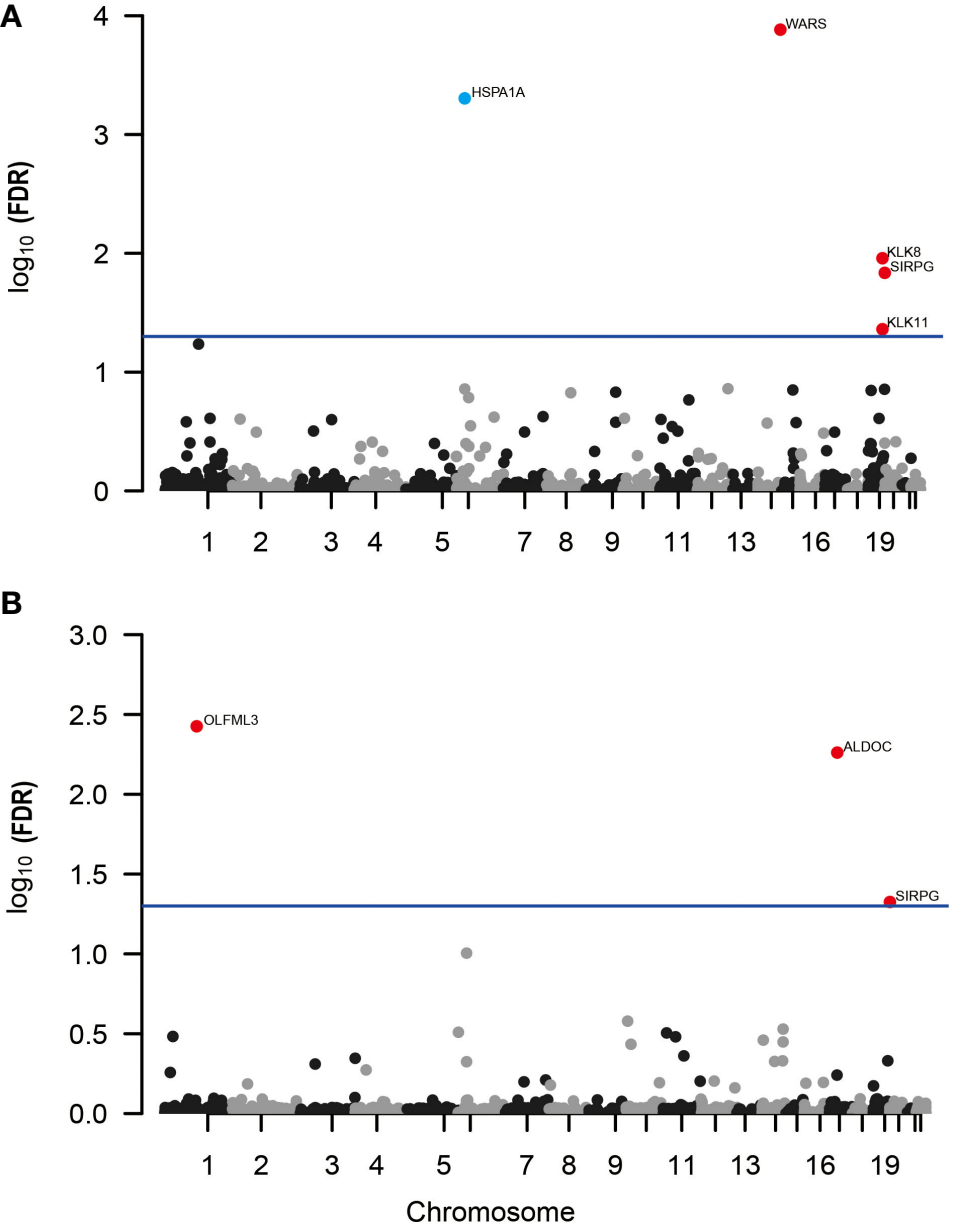
Manhattan plots for associations of genetically predicted 4657 plasma proteins levels with DR and PDR in MR analysis. **(A)** Associations of genetically predicted plasma protein levels with DR; **(B)** associations of genetically predicted plasma protein levels with PDR. Labelled and color genes refer to MR findings with FDR-corrected p < 0.05. Red genes indicate the positive effect of the plasma proteins on outcomes; blue genes indicate the negative effect of the plasma proteins on outcomes.

In our MR analysis, we assessed 4657 plasma proteins to explore their potential link with DR, employing a methodology previously explicated. After applying an adjustment for the FDR, we identified five proteins with significant associations with DR (**Figure 2A**). Specifically, we found that higher genetically predicted levels of the proteins WARS (OR = 1.16; 95% CI = 0.095-0.208, FDR = 1.31×10^-4^), KLK8 (OR = 1.22; 95% CI = 0.105-0.288, FDR = 1.10×10^-2^), SIRPG (OR = 1.15; 95% CI = 0.071-0.201, FDR = 1.46×10^-2^), and KLK11 (OR = 1.58; 95% CI = 0.222-0.696, FDR = 4.35×10^-2^) were associated with an increased risk of DR. In contrast, an increase in HSPA1A levels (OR = 0.466; 95% CI = -1.067 to -0.46, FDR = 4.96×10^-4^) was linked to a decreased risk of DR (**Supplementary Table 1**).

Further, when investigating PDR, our analysis revealed three proteins associated with a higher risk of developing PDR (**Figure 2B**; **Supplementary Table 2**). These include OLFML3 (OR = 1.51; 95% CI = 0.238-0.591, FDR = 3.75×10^-3^), ALDOC (OR = 1.56; 95% CI = 0.246-0.637, FDR = 5.48×10^-3^), and SIRPG (OR = 1.15; 95% CI = 0.068-0.208, FDR = 4.73×10^-2^). Notably, SIRPG showed a consistent association with both DR and PDR.

### 3.2 Replication analyses

In the replication phase of our study, we re-examined five proteins initially identified as associated with DR. Out of these, two proteins, WARS and SIRPG, showed consistent results in the Icelandic deCODE genetics study. The analysis indicated an increased risk of DR with higher levels of WARS (OR = 1.39; 95% CI = 0.21-0.45; p-value = 5.50 × 10^-8^) and SIRPG (OR = 1.39; 95% CI = 0.18-0.62; p-value = 3.32 × 10^-4^), as shown in **Supplementary Table 3**. Our sensitivity analysis, which included tests for heterogeneity (p-value for Cochran’s Q = 0.442) and pleiotropy (p-value for MR-Egger intercept = 0.479), found no significant discrepancies in the association between WARS and DR. It’s important to note that for SIRPG, due to the availability of only one IV (rs6043409), we couldn’t perform heterogeneity and pleiotropy tests (**Supplementary Table 4**).

For PDR, our analysis replicated the associations for all three identified proteins: OLFML3, ALDOC, and SIRPG. Specifically, increased levels of OLFML3 (OR = 1.82; 95% CI = 0.09-1.12; p-value = 2.19 × 10^-2^), ALDOC (OR = 1.83; 95% CI = 0.34-0.87; p-value = 9.28 × 10^-6^), and SIRPG (OR = 1.58; 95% CI = 0.23-0.68; p-value = 6.65 × 10^-5^) were associated with a higher risk of developing PDR. These results are detailed in **Supplementary Table 5**. Based on Cochran’s Q statistics, there was little evidence of heterogeneity between OLFML3 and PDR (p-value for Cochran’s Q = 0.108). Similar to SIRPG in DR analysis, due to the presence of only one IV for both SIRPG (rs6043409) and ALDOC (rs141921160), heterogeneity and pleiotropy analyses were not conducted.

### 3.3 Colocalization analyses

In our study, we conducted detailed colocalization analyses to further understand the relationship between certain plasma proteins and both DR and PDR. Our findings showed significant colocalization for three of the five analyzed proteins (KLK8, SIRPG, and WARS) with DR. The evidence for colocalization was strong, as indicated by the PPH4, which were 78.3%, 80.7%, 94.3%, and 87.6% respectively for these proteins (**Supplementary Table 6**). Similarly, for PDR, two proteins, ALDOC and SIRPG, also showed high evidence of colocalization with PPH4 values of 99.1% and 93.4%, respectively.

To classify the identified proteins based on the strength of evidence supporting their role in DR and PDR, we organized them into tiers. We considered several factors for this classification, including the consistency of results in replication analyses, the presence of heterogeneity or horizontal pleiotropy, and the strength of colocalization evidence. Based on this approach, WARS and SIRPG were categorized as Tier 1 evidence proteins for DR, indicating a strong link. For PDR, ALDOC and SIRPG were also classified as Tier 1 evidence proteins, underscoring their potential significance in the disease’s development. These categorizations and the detailed evidence supporting them are presented in **Table 1**.

**Table 1:** Summary of levels of evidence for target proteins for DR and PDR.

Disease	Protein	Discovery	Replication	Heterogenity	Pleiotropy	Colocalization	Targets
DR	WARS	1.31E-04	3.01E-07	4.42E-01	4.80E-01	94.4%	Tier 1 Target
	HSPA1A	4.96E-04	8.89E-01	1.45E-147	NA	0.0%	Tier 3 Target
	KLK8	1.10E-02	5.78E-01	1.45E-147	4.91E-02	78.3%	Tier 2 Target
	SIRPG	1.46E-02	3.32E-04	NA	NA	80.7%	Tier 1 Target
	KLK11	4.35E-02	4.94E-01	1.30E-01	6.01E-01	1.0%	Tier 3 Target
PDR	OLFML3	3.75E-03	2.19E-02	1.08E-01	NA	0.0%	Tier 3 Target
	ALDOC	5.48E-03	9.28E-06	NA	NA	99.2%	Tier 1 Target
	SIRPG	4.73E-02	6.65E-05	NA	NA	93.4%	Tier 1 Target

## 4 Discussion

In human genetics research, the focus on identifying disease biomarkers, especially for conditions like DR, is crucial. A large portion of FDA-approved drugs in recent years are supported by genetic research, highlighting the role of genetics in medical advancements (22). Our study, employing MR and colocalization analysis, identified four plasma proteins (WARS, KLK8, SIRPG, ALDOC) as potential markers for DR and PDR. Three of these proteins, WARS, SIRPG, and ALDOC, were validated in multiple MR analyses against an independent pQTL database. This validation strengthens our findings. Our study paves the way for further research to explore the direct histological links of these proteins to DR. It highlights the potential of these proteins as targets for future therapeutic interventions, given their relative ease of detection.

Our study highlights SIRPG as a significant marker for DR and its advanced form, PDR, supported by strong evidence. SIRPG, a member of the SIRP protein family, is primarily found on T cells and a subset of B cells (23). Genomic studies have linked two specific genetic variations of SIRPG, rs2281808 (C > T; intronic) and rs6043409 (G > A; A263 V), to type 1 diabetes (24, 25). The association of the T allele of rs2281808 with an increased risk of type 1 diabetes suggests a genetic predisposition (26). Further analysis using two-sample MR indicates a correlation between higher levels of SIRPG and an increased risk of type 1 diabetes (27). These findings suggest a potential link between elevated SIRPG levels and diabetes susceptibility. However, the direct role of SIRPG in the development and progression of DR and PDR needs further investigation. This research opens avenues for exploring SIRPG as a biomarker in diabetic eye diseases and understanding its underlying mechanisms in DR pathogenesis.

In our research, we identified WARS as another potential risk factor for DR. WARS, a fundamental enzyme in protein synthesis, links tryptophan to its corresponding transfer RNA (28). Previous studies have shown increased levels of WARS in the tears of patients with PDR, suggesting its involvement in the disease (29). Our findings support this link, establishing a causal relationship between WARS and DR. Interestingly, PDR is characterized by abnormal blood vessel growth, yet WARS is known to have anti-angiogenic effects. This includes its influence in ocular angiogenesis (30) and tumor growth (31). When WARS is broken down, it produces several smaller molecules, such as mini-WARS and T2-WARS, known to inhibit blood vessel growth (30–34). Specifically, T2-WARS interacts with VEGF pathways, reducing endothelial cell movement and angiogenesis (33, 35). Additionally, WARS acts as a natural trigger for Toll-like receptors (TLR) 2 and 4, known to play a role in inflammatory responses. The engagement of WARS with these receptors leads to the production of various inflammatory substances (36). Considering the known role of inflammation in DR, exploring the pathological implications of WARS in this context is of great importance (37). This further establishes WARS not only as a marker for DR but also as a potential target for understanding and managing the disease.

Our study highlighted ALDOC, a member of the class I fructose-bisphosphate aldolase gene family, as a key protein associated specifically with proliferative diabetic retinopathy (PDR). ALDOC’s primary function involves critical steps in glycolysis, where it aids in breaking down sugars (38). We observed that increased levels of ALDOC align with elevated plasma free fatty acid concentrations. This correlation could influence insulin secretion and potentially lead to type 2 diabetes mellitus, a known risk factor for PDR (39, 40).

Furthermore, research by Michal et al. has uncovered the significant role of aldolase proteins, including ALDOC, in enhancing Wnt signaling (41). This signaling pathway, implicated in various cellular processes, is critical in the development of diabetic retinopathy. Specifically, ALDOC and its family members can modify Wnt signaling by interacting with key molecular components, thereby influencing cell behavior related to PDR (42). Although these findings position ALDOC as a potential risk marker for PDR, its direct role in the disease’s pathogenesis is yet to be fully understood. More detailed studies are necessary to clarify the exact relationship between ALDOC levels and PDR, which could open new avenues for therapeutic interventions.

Our study has limitations, particularly in using blood-derived proteins, which may not completely reflect changes in DR-specific tissues like the retina. Future research should explore proteins from these ocular tissues for deeper insights into DR. Additionally, our use of SOMAmers technology, while advanced, may not capture the full range of proteins involved in DR. Exploring diverse proteomic methods and sample types could uncover more relevant proteins. Importantly, our focus on European ancestry limits the study’s broader applicability. Future research must include diverse populations to understand how genetic differences affect DR across ethnicities. This is crucial for developing treatments and prevention strategies effective for a global population.

In summary, our study identifies strong causal connections between three plasma proteins (SIRPG, WARS, ALDOC) and DR. This finding opens new avenues for therapeutic research in DR. Future studies are essential to confirm these links and explore their underlying mechanisms. This work sets the stage for developing targeted treatments for DR, addressing a significant health challenge.

## Data availability statement

The original contributions presented in the study are included in the article/**Supplementary Material**. Further inquiries can be directed to the corresponding author.

## Author contributions

XZ: Conceptualization, Data curation, Formal analysis, Writing – original draft. SY: Writing – original draft, Writing – review & editing, Formal analysis, Resources. YT: Project administration, Resources, Writing – review & editing.
